# Green technology innovation: Anatomy of exploration processes from a learning perspective

**DOI:** 10.1002/bse.2295

**Published:** 2019-05-03

**Authors:** Samuel Wicki, Erik G. Hansen

**Affiliations:** ^1^ Centre for Sustainability Management (CSM) Leuphana University of Lüneburg Lüneburg Germany; ^2^ Institute for Integrated Quality Design (IQD) Johannes Kepler University Linz (JKU) Linz Austria

**Keywords:** core competences, eco‐innovation, green innovation, green technology, innovation process, organizational failure, radical innovation, sustainability‐oriented innovation

## Abstract

This paper examines how established firms use their core competences to diversify their business by exploring and ultimately developing green technologies. In contrast to start‐ups dedicated to a green mission, diversifying into green markets by developing new products based on existing core competences has proven to be challenging. This is because the exploration processes to find a match between green technology opportunities and internal competences is complex and new to most established firms. This paper gains insights into exploration processes for green technologies and the learning modes and outcomes linked to these processes. We examined exploration processes at the microlevel in an embedded case study of an engineering firm using a combination of the “fireworks” innovation process model and organizational learning theory. First, we found that developing green technologies involves a long‐term exploratory process without guarantee of (quick) success and likely involves many exploration failures. Second, as exploration unfolds along multiple technology trajectories, learning occurs in individual exploration paths (on‐path), when new paths are pursued (path‐initiation), and when knowledge from one path is spilled over to subsequent paths (across‐paths). Third, to increase their chances for success, firms can increase the efficiency of exploration by fostering a failure‐friendly organizational culture, deliberately experimenting, and purposefully learning from failures.

## 1 INTRODUCTION

Despite important literature on why firms become more sustainable and what it means for a firm to be sustainable, there is still limited literature on how firms transform themselves in order to become more sustainable (Zollo, Cennamo, & Neumann, 2013). Relatedly, the literature on eco, green, or sustainability‐oriented innovations has grown considerably (Hansen et al. 2009; Schiederig, Tietze, & Herstatt, 2012), but the innovation processes of how organizations are “going green” is still only marginally understood (Klewitz & Hansen, 2014). This is particularly the case for established firms previously offering conventional product portfolios that then aim to diversify into green markets—in contrast to entrepreneurial firms founded with a green mission. We are therefore interested in how established firms search for sustainability‐related business opportunities, develop green technologies, and exploit niche markets (Hart, Milstein, & Caggiano, 2003; Jenkins, 2009; Schaltegger, Lüdeke‐Freund, & Hansen, 2012). For established firms, green technology innovations are often radical as they significantly differ from their current business in terms of technology and markets, and involve large unknowns (Driessen, Hillebrand, Kok, & Verhallen, 2013).

As green innovation processes are new to many established firms, adapting to green technology as well as product and market contexts requires important learning efforts (Siebenhüner & Arnold, 2007), a process that is presently not well understood but is thought to be hindered by past beliefs about business success. What is also often neglected is the role of trial and error as well as failures in innovation processes (Cannon & Edmondson, 2005; Khanna, Guler, & Nerkar, 2016; Sitkin, 1992). Therefore, this paper aims at understanding how green technology innovation processes unfold in established organizations, and what the role of learning from failures plays. To do so, we investigated microlevel learning processes in a business‐to‐business engineering firm previously operating solely in conventional markets, but later also engaging in radical innovation targeted at green markets, particularly sustainable energy technologies (SETs). Focusing on the early phases of the innovation process and less on product development and diffusion, we examined how the firm explored green technology and related markets. We analyzed these outcomes from an organizational learning perspective (Crossan, Maurer, & White, 2011; Lozano, 2014) with a focus on learning from failures (Cannon & Edmondson, 2005; Khanna et al., 2016; Sitkin, 1992).

We contribute to the literature on green innovation in three ways: (a) by developing a path‐based learning framework for green technology innovation involving multiple paths, emerging branches, and dead ends. (b) By showing that learning occurs at three points in time: during an existing path (on‐path), when new path branches are opened (path‐initiation), and finally when the experience of multiple paths is considered (across‐paths). The path‐based view also shows the inherent complexity of green technology innovation. (c) By discussing two key innovation practices—deliberate failures and intelligent trial and error—that can be managed to increase chances for success.

The remainder of this paper is structured as follows: Chapter 2 reviews the literature on green innovation and organizational learning. Chapter 3 presents the methodology and introduces the embedded case study. Chapter 4 analyses the learning outcomes, and Chapter 5 concludes the paper by discussing how a conventional firm can explore green technologies and markets and how this exploration can be managed.

## 2 LITERATURE REVIEW

### 2.1 Green technology innovation processes

Green innovations include new technologies, products, services, or business models that have positive impacts on the environment and society (Adams, Jeanrenaud, Bessant, Denyer, & Overy, 2016; Foster & Green, 2000; Seebode, Jeanrenaud, & Bessant, 2012) or fulfil the needs of customers with lesser harmful impacts than the alternatives (Goodman, Korsunova, & Halme, 2017). Compared with conventional innovations, they share many similarities but differ strongly in purpose, complexity, direction of search, and uncertainty (Bos‐Brouwers, 2009; Klewitz & Hansen, 2014; Noci & Verganti, 1999; see also Dangelico, 2016 for a review of green product innovation). Indeed, in addition to commercial success, green innovation embraces the explicit dual aim of improving the firm's sustainability performance and contributing to solving societal problems (Hansen, Große‐Dunker, & Reichwald, 2009; Hart et al., 2003), thus helping firms to become sustainable development agents (Scheyvens, Banks, & Hughes, 2016). Firms need to search for innovation in a specific direction to assure that the outcomes will have positive impacts on sustainability.

A common way for established firms who aim to develop green technologies is to engage in a diversification process. Diversification allows existing business to be complemented with green technologies, following what Hart (1997) calls a clean technology strategy (see also Jenkins, 2009, on competitive advantage through green product innovation and unserved markets). We focus on resource‐based (Ansoff, 1957; Montgomery, 1994) diversification, which involves using the existing resources and core competences (Prahalad & Hamel, 1990) a firm already has to enter new green (niche) markets.

There is important literature on the determinants of innovation and innovation outcomes (Díaz‐García, González‐Moreno, & Sáez‐Martínez, 2015; Kiefer, Del Río González, & Carrillo‐Hermosilla, 2018), for instance, discussing the importance of innovation for long‐term survival (March, 1991) and drivers and barriers to developing innovations (Tidd & Bessant 2009), also in the context of Sustainability‐oriented Innovation (SOI) (Álvarez Jaramillo, Zartha Sossa, & Orozco Mendoza, 2018; Bos‐Brouwers, 2009). However, the innovation process perspective is underdeveloped in the literature (Crossan & Apaydin 2010), and even more so in the SOI context. This process perspective can be further developed based on several existing innovation process models (Verworn & Herstatt 2002).

The fireworks model allows to study innovation processes, which refer to sequences of activities that lead to the birth of an innovation (Crossan & Apaydin 2010). So called flow‐models are often used to study innovation processes (Verworn & Herstatt 2000). Frequently used in innovation management and in the design literature, these models represent the archetypical development of an innovation in the form of a linear process that ranges from the idea to the launch of the new product (Verworn & Herstatt 2000). This prescriptive view yields limited usefulness for empirical analysis. Indeed, in reality, innovation processes are often complex and rather chaotic, particularly in early phases or in radical innovations (Koen et al., 2002). Based on an in‐depth qualitative study of 30 British industrial firms known to be active in research and development, Cooper (1983, 12) concludes that

[T]he new product process is not the sequential or series process so often portrayed in the literature. Rather, we see a more complex process, with many activities overlapping or undertaken in parallel. Indeed there appear to be certain efficiencies in adopting this parallel approach. The usual normative models, in contrast, propose a stagewise (series) set of activities for new product managers to follow. Such models are clearly unrealistic: product innovation simply does not occur that way, and normative guides that do not recognize either the differences in processes or the overlapping nature of activities will probably meet with little success.

**Figure 1:**
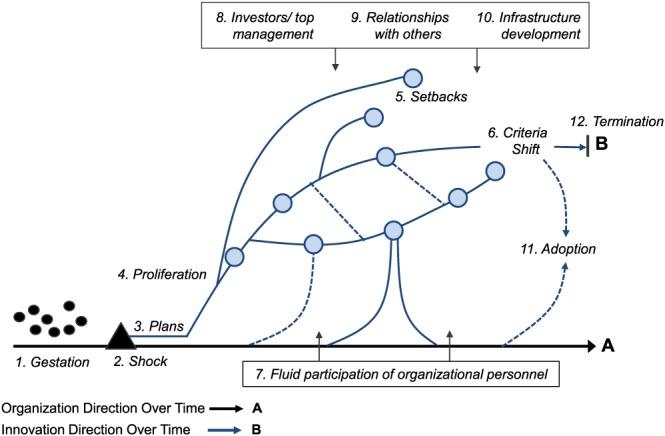
The innovation journey and its key components (Van de Ven et al., 2008) [Colour figure can be viewed at wileyonlinelibrary.com]

Few analytical models embrace the complexity of the innovation process described by Cooper. This explains why this research uses the fireworks innovation process model which allows to study green technology innovation processes without reducing their complexity. The model was already successfully used in German literature to examine cases of sustainability innovation (Fichter et al. 2007). It is depicted in Figure 1 (Van de Ven et al. 2000/1989). A full description can be found in Van de Ven, Polley, Garud, and Venkataraman (2008).

The fireworks model suggests that these innovation processes, taken together, form an innovation journey that typically begins with a long time period where ideas are in gestation. The actual journey begins with a shock that signals the urgency to develop innovations to guarantee the survival of the organization (March, 1991). After the shock, new product ideas start proliferating as different innovation paths (curved lines). In the case of green technology, each path typically relates with a different combination of technology and market and has many activities to explore if this combination can lead to a commercially successful new product. However, as the literature indicates, by far, not all paths lead to success, which often alternate with failures (Van de Ven et al., 2000/1989; Maidique & Zirger, 1985).

### 2.2 Organizational learning and learning from failures

The organizational learning literature discusses how firms learn to adapt to new business environments and develop new innovations (see reviews by Dodgson, 1993; Crossan et al., 2011; also in the context of green technology innovation, Siebenhüner & Arnold, 2007). Organizational learning can take various forms and involve multiple processes. Many authors use the well‐established behavioral psychology concepts of single and double‐loop learning (Argyris & Schön, 1978). In single‐loop learning, a mistake is corrected by using a different action to attain the same goal. Although the set of actions changes, the goal remains the same. Double‐loop learning is a more complex process in which the mistake is corrected by rethinking the original goal. A person in the process of learning will not only reflect on and change their actions to attain the goal, but also change the goal itself. The new set of actions will therefore be aligned with the reevaluated goal. Double‐loop learning also involves an organization questioning its underlying norms, mental frames, world‐views, sets of beliefs, routines, and assumptions about success (Tripsas & Gavetti, 2000).

Although the literature rarely indicates whether learning is induced by successes or failures (Khanna et al., 2016), as exploration is typically punctuated by failures, we pay particular attention to the latter. Learning from failures has received much less attention (Cannon & Edmondson, 2005), but several authors argue it is important as failures provide more valuable feedback (Sitkin, 1992). These authors maintain that small failures are essential prerequisites for effective organizational learning and encourage deliberate experimentation to trigger learning from failures (Khanna et al., 2016).

**Figure 2:**
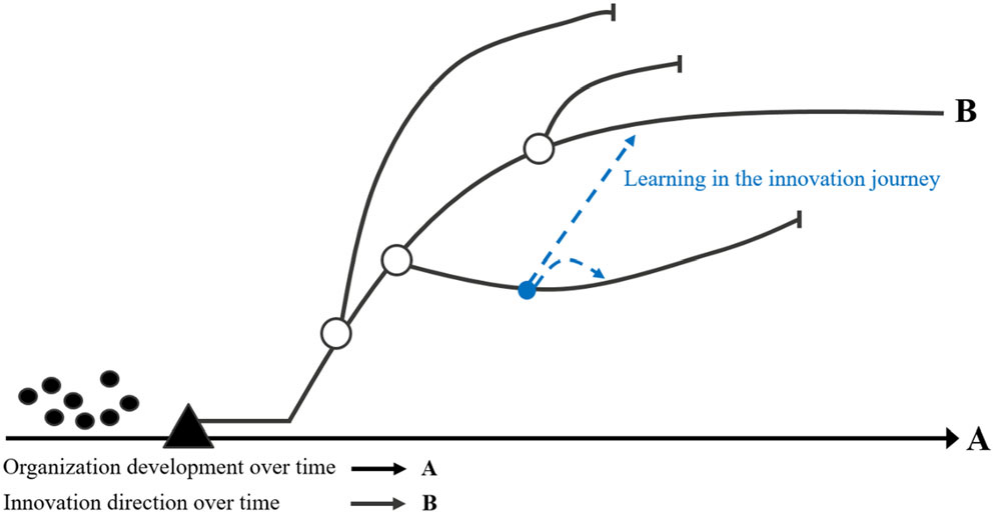
Preliminary conceptual framework: the fireworks innovation process model (Van de Ven et al., 2008) combined with organizational learning [Colour figure can be viewed at wileyonlinelibrary.com]

Overall, our understanding of green technology innovation processes, nonlinear innovation processes (taken from the fireworks innovation model with its emphasis on trial and error), and organizational learning are used to build our preliminary conceptual framework (see Figure 2). In this framework, the innovation paths (curved black lines) represent a specific exploration of a new product idea intended for a new market. In each path, the organization (further) develops green technologies or their components — with more or less distance to its existing core technologies — with the aim to commercialize them as new products for new markets. This exploration generates learning outcomes for green innovation (Hoffmann, 2007) that may be useful on subsequent innovation paths (symbolized by blue arrows).

## 3 METHODOLOGY

### 3.1 Research design

The aim of this paper is to investigate how green technology innovation processes unfold in organizations and what can be learned from exploration failures. To address this goal, we undertook an in‐depth longitudinal and embedded case study (Yin, 2014) in a well‐established German engineering firm successfully operating in the electronics industry. We specifically focus on the learning outcomes that occurred within a new business line dedicated to the exploration of SETs. Although we are interested in understanding the overall innovation process, to obtain deep insights into the process, the research features four embedded cases corresponding to innovation paths focused on specific products and related technologies. We follow other innovation scholars who adopt this method and focus on several embedded cases of unsuccessful innovation (for example, Cannon & Edmondson, 2005; Khanna et al., 2016; Tripsas & Gavetti, 2000).

### 3.2 Case selection

#### 3.2.1 Overview

We conducted theoretical sampling (Eisenhardt, 1989, 537) with the aim of extending the literature on green technology innovation. The exploratory case chosen is critical for expanding this body of knowledge (Yin, 2014, 41) in four directions as follows:

1. First, instead of a company founded with a green mission (Schaltegger & Wagner, 2011), we searched for an established firm with conventional product portfolios that was developing innovations in unknown green markets—hence, representing “green diversification.” This phenomenon has not been examined yet (Jenkins, 2009; Klewitz & Hansen, 2014).
2. Second, although learning from failures has been recently receiving more attention in the organizational learning literature (Baumard & Starbuck, 2005; Cannon & Edmondson, 2005; Khanna et al., 2016), empirical studies about failures are still sparse. Furthermore, to our knowledge, the case of a firm learning from failures in innovation processes for radical innovation has not been researched yet, even though failures are frequent in this context.
3. Third, fine‐grained studies of innovation dynamics at the microlevel have seldom been reported in the green technology innovation literature (Schiederig et al., 2012; Zollo et al., 2013).
4. Finally, TechLtd as a small and medium-sized enterprise (SME) is a good representative for radical innovation in resource‐constrained contexts. In their systematic literature review, Klewitz and Hansen (2014) found that some SMEs have very proactive approaches toward sustainability. Proactive SMEs are more inclined to develop radical innovations and search to solve sustainability problems in an entrepreneurial way (Aragón‐Correa, Hurtado‐Torres, Sharma, & Garcia‐Morales, 2008), even those operating in a resource constrained environment (Halme & Korpela, 2014). The results are likely also applicable to larger more resourceful entrepreneurial firms.

The case is revelatory (Yin, 2014) because it is difficult to obtain (longitudinal) access to unfolding innovation processes, particularly at an early stage. Indeed, early stage processes are difficult to identify and study as they do not always lead to successful innovation outcomes. This is even more true for unsuccessful innovation paths, which potentially weaken an organization's (or individual manager's) reputation as a “successful innovator.” In fact, this study was only possible through an engaged scholarship approach involving the development of close ties with the organization's top management prior to the research project (van de Ven, 2007).

#### 3.2.2 Introducing TechLtd

This paper examines TechLtd, a medium‐sized German engineering firm operating in business‐to‐business markets in the electronics industry. The family business, founded in 1962 and owner‐managed in the second generation, employs about 200 people. Over the past 50 years, it has accumulated extensive knowledge in control systems for high‐speed engines and generators, and has become a global leader with a market share of about 40% in its main market, machine‐tools for circuit‐board drilling. It has the typical characteristics of a “hidden champion” (Simon, 2009).

TechLtd develops and produces electronic components (computerized numerical control system and control systems for high‐speed motors and generators) that are sold to manufacturers of machine tools (its primary market), turbines, or various other industrial machine manufacturers. Product development typically takes several months and is characterized by intensive research and development collaboration with customers and trust‐based, long‐term relationships. Production is typically done in small batches and is, contrary to industry trends, fully located in Germany. Sales offices exist in Europe, the United States, and Asia. Top management, knowing that new path‐breaking technologies might weaken its main market segment (representing 80% of sales) sometime in the future, recognized the urgency for exploring new product and market areas. Inspired by their intrinsic motivation for sustainability (Baumard & Starbuck, 2005), top management sought new applications for the core technologies that underlie in their existing products (Taylor & Helfat, 2009) and found several in emerging SET markets. After a long gestation period, a new business line dedicated to the exploration and development of SET‐related technologies and markets was created.

**Figure 3:**
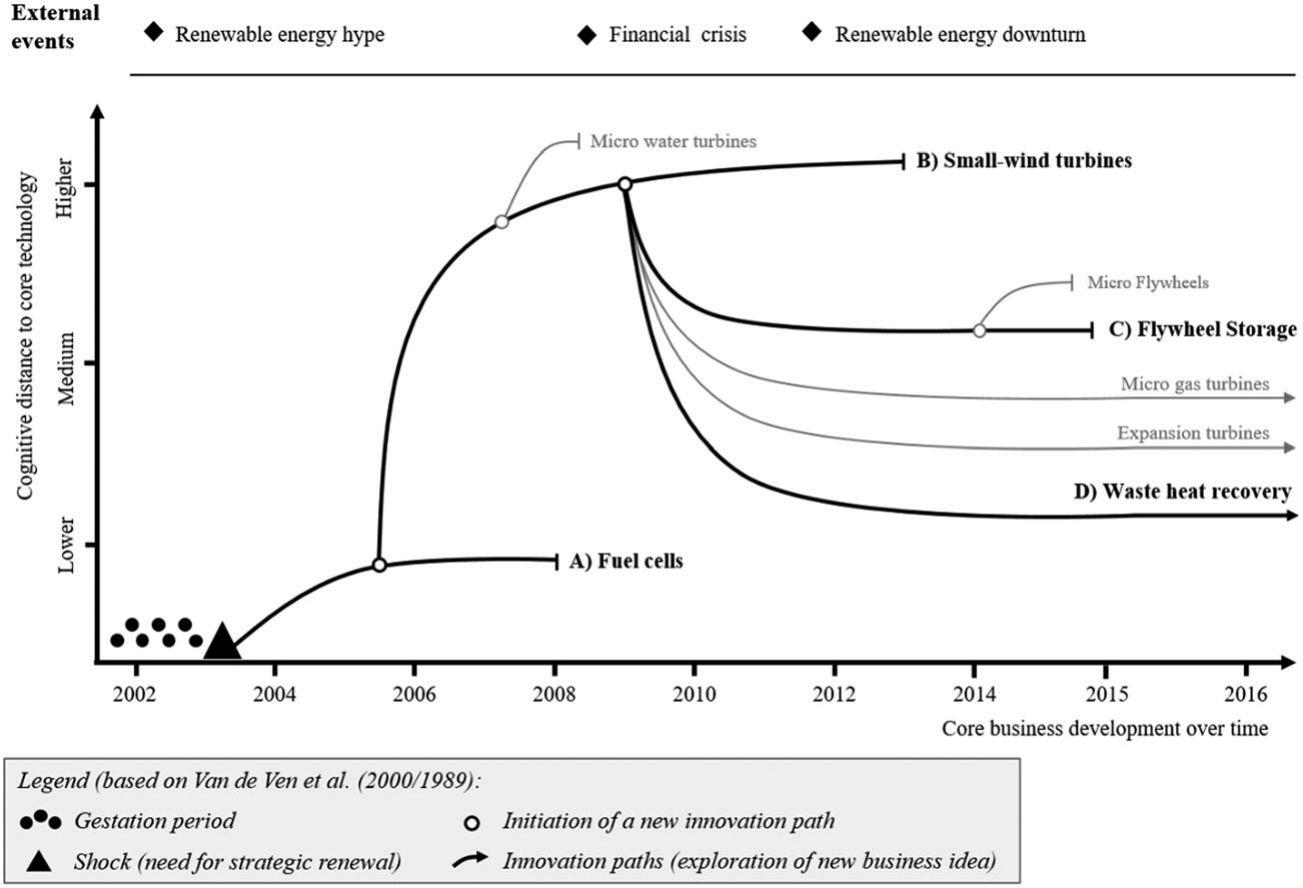
Innovation paths representing the exploration of new business ideas at TechLtd

The focus of this paper is on this new business line, called feed‐in technology, which is characterized by an exploration rationale. It was formally created in 2003 to explore how the company could use its engineering competences (and related core technologies) to develop new applications for the market of renewable energy technologies (particularly small wind turbines; see Wicki, 2015). An engineer was hired externally to lead the exploration and began to search for synergies between these existing core competences and applications in the area of rotation‐based SET, mostly different forms of turbines. Exploration led to the four main innovation paths (P1–P4), illustrated in Figure 3 and described in Table 1: fuel cells (P1), small wind turbines (P2), flywheel energy storage (P3), and waste heat recovery (P4). An overview of the paths and an analysis of their individual learning outcomes can be found in Section 4.

**Table 1:** Descriptive overview of the innovation paths

**Path characteristics**	**P1: Fuel cells (FC)**	**P2: Small wind turbines (SWTs)**	**P3: Flywheel storage (FS)**	**P4: Waste heart recovery (WHR)**
Overview	Used core technology to develop control system for high‐speed turbines supplying FCs with combustion gases. Technology development with a large automaker that was control system customer	Developed controller for SWTs with partner firm with knowledge about and access to small wind market (met on Path P1). Relied on existing core technology and new components from previous path.	Explored high‐speed flywheels (kinetic energy storage devices). Flywheel controller based on the SWT controller, without turbine management functions, and customized to flywheel application. Many technological components reused.	Used controller in ORCs turbines for the recovery of low temperature heat. Controller based on SWT controller and adapted to needs of gas turbines.
Related energy technology	FCs	SWTs	High‐speed FS (energy storage)	Industrial WHR
Product (component) description	Controller for turbine supplying combustion air to FCs. Controller equipped with inverter for grid feed‐in of generated electricity.	Controller for SWT (<10 kW), turbine management, and grid feed‐in of generated electricity	Controller for high‐speed flywheels storing kinetic energy over short time periods (<1 day).	Controller for gas turbines in WHR based on the ORC principle.
Duration	5 years (2003–2008)	8 years (2005–2013)	4 years (2010–2014)	Ongoing (2010 onwards)
Staff expenses	<0.5 million euros	3–4 million euros	<0.5 million euros	<0.5 million euros
Technology	High‐speed (HS) drive electronics (core business), grid feed‐in technology	HS drive electronics, grid feed‐in, and turbine management	HS drive electronics and grid feed‐in	HS drive electronics and grid feed‐in
Market description	Automotive market (cars), FC for decentralized electricity production	Decentralized energy production to increase energy autarky and reduce energy costs in households, agriculture, and industry	Short‐term electricity storage for grid stabilization, control power, Uninterrupted power supply (UPS) and home storage. Flywheels can be used to recover braking energy in vehicles.	Heat recovery from low temperature sources such as (industrial) waste heat and geothermal sources.
Upfront market exploration	Very limited (outsourced to main customer)	Medium (relied on business partner); Exploration toward the end only	Important	Important
Exploration steps and related activities	1. Discovers FC thanks to R&D project of large automaker 2. Adapts existing controller to FC turbines and produces 20 prototypes 3. Terminates path	1. Discovers SWT thanks to university spin‐off met on P1 and discusses potential to develop inverter 2. Begins partnership with spin‐off 3. Market analysis focusing national grid feed‐in requirements and regulations 4. Builds several prototypes 5. Tests prototypes with potential customers 6. Builds a product and engineers an improved version (V2) 7. Advertises product at trade fairs and in industry press 8. Begins small series production 9. Launches product 10. Market analysis of other countries to understand why sales not increasing 11. Strengthens team for final sales effort 12. Terminates path and exits market	1. Discovers FS thanks to previous partnership with university 2. Builds prototype based on product developed in P2 (removes some components and adapts to FS) 3. Searches for other potential customers than the university 4. Approaches end user to initiate joint product‐development project. 5. Approaches market leader in the United States to study its business model 6. Attends industry workshop 7. Terminates path due to negative market outlook	1. Discovers controller can be also be used for WHR applications 2. Studies regulatory context 3. Based on prototypes of P2 and P3, builds product for WHR applications 4. Searches for potential customers and sells them custom‐made product 5. Adopts wait‐and‐see approach, given uncertain market outlooks
Rationale for path initiation	Promising future technology with immense market potential Automotive market seen as strategically important Very high importance for sustainability (new high‐efficiency renewable energy generation technology)	Niche markets fits existing production capability Challenging engineering tasks. Secure large market shares through mastery of HS technology and gain unique competitive advantage Large contribution to sustainability by facilitating diffusion of SWTs	Minor R&D development costs (largely same technology as SWT) Niche markets fits production capacity Relevance for sustainability (energy storage)	Minor R&D development costs (largely same technology as SWT) Niche markets fits production capacity Relevance for sustainability (increase energy efficiency)
Rationale for continuing or path termination	Terminated as FC technology not mature for commercial applications Market size of consumer cars does not fit niche strategy	Terminated due to poor sales	Wait‐and‐see as technology not mature for commercial applications End‐user business model not financially viable	No termination, but wait and see because markets still emerging and unpredictable
Sustainability ambition	High: new generation of high‐efficiency energy conversion technology	High: new energy conversion technology. Provision of missing piece in technology diffusion: the energy inverter	Medium: short‐term storage to increase system efficiency and support renewable energy diffusion. Energy efficiency increases of up to 35%	Low: limited system efficiency increase, mainly due to energy recovery in industrial processes

*Note*. FC: fuel cell; HS: high‐speed; ORC: organic Rankine cycle; SWT: small wind turbine; R&D: research and development; UPS: uninterrupted power supply; WHR: waste heat recovery.

### 3.3 Data collection

We utilized a combination of retrospective and real‐time approaches (Pettigrew, 1990) covering a period of 15 years (2000–2015), of which we were able to observe the last three as an on‐going process (2013–2015). To assure construct validity (Babbie, 2013), we triangulated various data sources (Table 2) including formal semistructured interviews with top management, middle management, and value network actors; informal and unstructured interviews (e.g., informal conversations in the target company), participatory observation, and focus groups at top‐management meetings; observation of industry workshops; and extensive desk research. We also conducted action research (Huxham & Vangen, 2003) and took the role of a facilitator in some of the innovation paths. For instance, we organized a flywheel innovation workshop with current and potential partners of the focal company. The interviews were transcribed, other data (e.g., site visits, participant observation, focus groups) were protocolled (Babbie, 2013), and both were coded using software for qualitative data analysis (MAXQDA).

**Table 2:** Data collection methods

Data types	Sources		
	Internal: top and middle management	External: business partners and value chain actors	Total
Semistructured interviews	8 interviews	21 interviews	29
Informal unstructured interviews	3 interviews	18 interviews	21
Focus group sessions	3 sessions	n/a	3
Participant observation	3 meetings	2 industry events	5
Action research	Seven AR events	3 AR events, including one major industry event organized (flywheel workshop)	10
Document analysis	25 internal documents (e.g., market studies, sales statistics, and customer lists)	Over 300 publicly available documents (e.g., industry reports, market analyses, newspaper and magazine articles, and websites of industry actors)	300+

### 3.4 Data analysis

**Figure 4:**
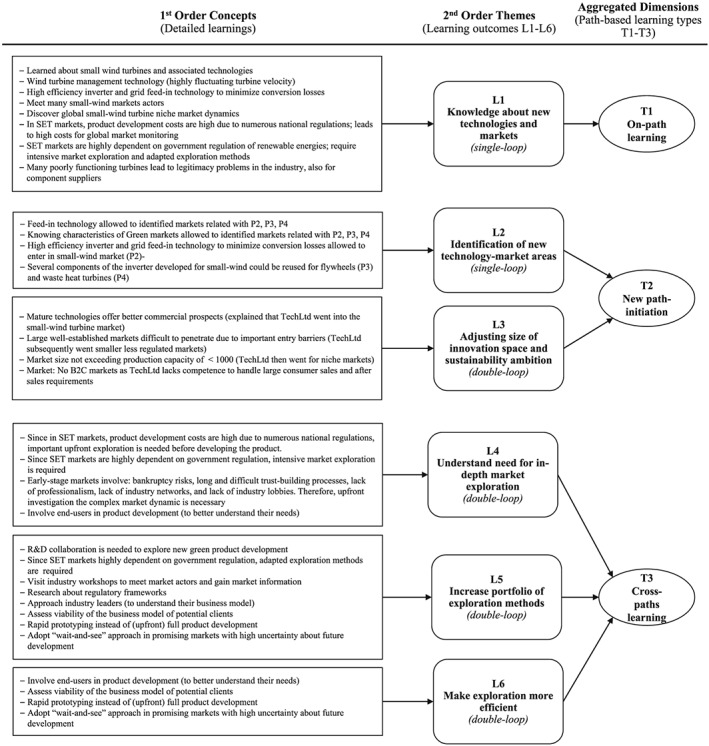
Data structure based on the Gioia methodology: path‐based learning types

In line with recommendations for longitudinal case studies (Huber & van de Ven, 1995; Van de Ven & Poole, 1990a; Yin, 2014), we started the analysis by reconstructing the timeline of the innovation process (Wicki 2015; Wicki, Hansen, & Schaltegger, 2015). Specifically, we analyzed (temporal) events along the innovation trajectory such as setbacks, changes in search direction, fluid participation of personnel, involvement of top management, evolution in success metrics, cognitive representations, beliefs, world‐views, and routines. These events were observed by tracking ideas, people, transactions, contextual events, and outcomes, following the fireworks innovation process model (van de Ven & Poole, 1990a). We referred to the fireworks model for longitudinal analysis (Poole & van de Ven, 1989) because it allows rich analysis of complex nonlinear processes on the microlevel. The analytical process involved three iterative coding steps, comparable with the recommendation of Gioia, Corley, and Hamilton (2012) for inductive research. First, our empirical data was coded into first‐order concepts (detailed learning outcomes, see Table A1) for each innovation path. Second, the data was aggregated into second‐order themes (learning outcomes Learning 1–6). Third, we distilled the themes into aggregated dimensions (path‐based learning types T1–T3). Figure 4 provides a graphical overview of the data structure, with the detailed learnings of the small wind turbines path (for consistency reasons). Table A1 provides the full range of learnings as they related to all four innovation paths (P1–P4).

We assured “trustworthiness” (Shenton, 2004) by addressing the criteria of credibility (internal validity), transferability (external validity), dependability (reliability), and confirmability (objectivity) with various research strategies such as triangulation of data types and informants, multiple investigators, transparency of the methodological approach, rich description of the phenomena and context, as well as consideration of alternative explanations.

## 4 RESULTS

**Figure 5:**
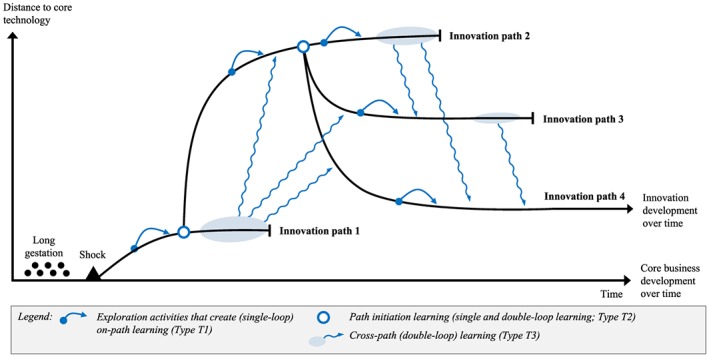
A path‐based learning framework for green technology innovation (based on (Van de Ven et al., 2008) [Colour figure can be viewed at wileyonlinelibrary.com]

Given the longitudinal character of our research, the study design with a long time frame, and our emphasis on learning from failures, this section analyses the learning outcomes as they relate to the four innovation paths (Figure 5; Table 3; Table A1). The results shows innovation paths that represent green technology exploration processes of new products, new markets, or a new combination of product and market (i.e. a “pure exploration” according to Voss & Voss, 2013). The vertical axis indicates the cognitive distance to the original core technology (Li, Vanhaverbeke, & Schoenmakers, 2008). The innovation paths are punctuated by exploration activities that aim at determining whether the new technology market area is interesting for the firm, whether commercial viable products can be developed, and, if they can, how the new product can be developed and commercialized. Each path consists of numerous small continuous changes (incremental innovations), whereas the emergence of a new path results from a (discontinuous) change in the technology‐market idea. In the most successful case, a path ends with the commercialization of a new product.

**Table 3:** A path‐based learning framework for green technology innovation

Learning dimensions	Learning types and their relation to innovation paths		
	T1: on‐path learning	T2: path‐initiation learning	T3: cross‐paths learning
Timing	Early	Middle; during process	Overall; toward process end
Learning mode	Single‐loop	Single and double‐loop	Double‐loop
Learning outcomes	Learning 1: Knowledge about new technologies and markets	Learning 2: Identification of new technology‐market areas (single‐loop) Learning 3: Adjusting size of innovation space and sustainability ambition (double‐loop)	Learning 4: Understand need for in‐depth market exploration Learning 5: Increase portfolio of exploration methods Learning 6: Make exploration more efficient
Sustainability aspects	Specific characteristics of green technologies and related markets; importance of the firm's core competence for sustainability	Identify related green technologies, contributing to the market area Trade‐off between wide innovation space and high sustainability ambition Gain more realistic perspective on potential (technological) contributions, also for commercialization	Learn to explore green markets (with complex dynamics) and cope with high cognitive distance between existing conventional and new green market areas

The analysis reveals that (single and double) loop learning outcomes relate to the innovation paths in three ways (Table 3): first, as outcomes on the path that are directly useful for the innovation processes in this same path (T1); second, as outcomes that initiate new paths (T2); third, as outcomes that improve overall innovation performance at a metalevel across paths (T3). The subsections below discuss these three innovation types (T1–T3) and the related learning outcomes (Learning 1–6).

### 4.1 On‐path learning (T1)

#### 4.1.1 Knowledge about new technologies and markets (Learning 1)

Even though not all innovation paths led to successful commercialization, they generated important knowledge about new green technologies and markets. As will be later analyzed in detail, it is this on‐path learning which also becomes useful to exploration of subsequent innovation paths (see T2). First, the firm added new technologies to its technological repertoire. On path P1, it discovered the feed‐in technology that allowed it to transform renewable energy to fit grid characteristics (Table 1). This component proved to be useful for all paths. TechLtd also met new actors. For instance, the partner of the small wind turbine controller (on path P2) was initially met on the first path P1. Furthermore, it discovered how it could best make use of its core competences in these green markets. Top management had a “eureka moment” when it realized that its high‐speed generator control competences could be used to solve an important problem in the emerging small wind turbine industry: the absence of an (efficient) inverter customized to small wind applications (Wicki, 2015).

Second, on each path, the firm also learned about new market environments (Table 1): the automotive fuel cell on Path P1, the small wind turbine on P2, the flywheel on P3, and finally waste heat recovery on P4. In addition to discovering new markets, it learned about the specific characteristics of green markets, which at an early stage, typically have very different dynamics than the mature markets with which TechLtd was familiar. Most of the market‐related learning occurred toward the end of Path P2, when TechLtd began to actively explore the small wind market itself. This market is illustrative of an emergent green market as participation is very volatile, with many small wind turbine manufacturers entering and leaving the market or going bankrupt, often due to a lack of professionalism. At the market level, the lack of an industry association, producer networks, or lobbying organizations revealed a low level of market formalization.

### 4.2 Path‐initiation learning (T2)

Learning outcomes allowed the firm to set out on new innovation paths that would not have been considered if they had not explored previous paths. In this regard, two learning outcomes played an important role: first, the identification of new technology‐market areas; (Learning 2) and second, the adjustment of the size of the innovation space (Learning 3).

#### 4.2.1 Identification of new technology‐market areas (Learning 2)

One of the most striking findings is that the exploration of a technology‐market area allows the firm to identify other—different but still related—green technology‐market areas and can thus open subsequent innovation paths. It appears that the discovery of new areas is path‐dependent on the technology and market knowledge gathered on the earlier paths (Learning 1). Consequently, even if a path leads to a setback, the discoveries made can allow a firm to identify new and promising paths. For instance, discovering how the automotive market (P1) works (for example, the stringent safety requirements) allowed TechLtd to recognize the market opportunity of flywheels as onboard storage in heavy‐duty vehicles (P3). In fact, as explained in Learning 1, the feed‐in technology discovered on Path P1 allowed the firm to enter other SET markets. Exploration on all subsequent paths was thus conditional on having embarked on path P1 (Table 1).

#### 4.2.2 Adjusting the size of the innovation space (Learning 3)

The size of the innovation space—the area in which a firm search for new innovation opportunities—adjusts over time as the firm learns more about which green technology‐market area fits its assets, competences, and resource base. As it became progressively less constrained by its old business model—and related cognitive frames—the firm developed a better sense of what innovations were feasible and realistic. This is important for two reasons: first, better knowing what is feasible reduces the risks of initiating an innovation path that appears promising at first sight but is not realistic; second, it prevents missing out on viable innovation opportunities that the firm would not see because it had considered them as unrealistic. The data shows how this space was both widened and narrowed over time (Table 1), and how eventually a trade‐off between a wide innovation space and high sustainability ambition was found (see also Wicki et al. 2015).

Sustainability guided TechLtd to technology‐market areas that they had not previously considered. In this sense, sustainability widened the innovation space. At the beginning, top management hoped to have a very high positive impact and was consequently only interested in highly innovative energy generation technologies such as fuel cells. Following this rationale, it aimed to contribute to what it perceived as high impact technologies: at first, fuel cells (P1) and later small wind turbines (P2). This selection significantly narrowed down the innovation space. When it experienced difficulties penetrating these markets, TechLtd again widened the space from alternative energy generation (P1 and P2) to energy storage (P3), and finally even more broadly to energy‐efficient technologies (P4). Although the innovation space was widened in this second phase, the decision to develop components that merely increased the efficiency of existing technologies (rather than engaging with high‐impact innovative technologies) implies that TechLtd's initial ambition to develop sustainable technologies strongly decreased, and hence its potential sustainability impact. Thus, over time, the firm became more realistic with regard to its level of sustainability ambition.

### 4.3 Cross‐path learning (T3)

In addition to learning how to initiate new paths, double‐loop learning also allowed the firm to reflect how exploration is done and draw important lessons for future innovation management practice. This reflection at the metalevel involved three learning outcomes. First, the firm came to understand the need for in‐depth market exploration (Learning 4). Second, it increased its portfolio of exploration methods and became proficient at selecting the appropriate one (Learning 5). Third, it learned to make exploration more efficient (Learning 6).

#### 4.3.1 Understanding the need for in‐depth market exploration (Learning 4)

The firm learned across paths about the importance of market exploration—including end users—in assessing the validity of new business ideas before significantly committing resources. In‐depth market exploration is particularly important in the context of emerging technologies with poorly established markets, a situation typical in the renewable energy context (see also Learning 1). Indeed, the dynamics of early‐stage markets are more complex (Suurs, Hekkert, Kieboom, & Smits, 2010) and thus require in‐depth exploration.

The findings show progressive learning about the importance of market exploration across the four innovation paths (see Table 1). On the first path (P1), TechLtd simply delegated market exploration to its main OEM customer in the belief that this major automotive player would be familiar with industry trends, trusting its interpretation and simply waiting until its own order book would fill up. Following this rationale, TechLtd did not seek to acquire any market information on its own. On path P2, exploration was largely delegated to a business partner with prior knowledge of the small wind market. In fact, its market knowledge was a major reason for initiating the joint venture. TechLtd began exploring the market with maladapted core business methods only toward the end of the path when sales did not increase. It is only when TechLtd had come to realize the importance of market exploration and when it no longer trusted the assumptions of its business partner that they began to carry out its own in‐depth market exploration. An important change happened from path P3 onwards: it systematically began to explore markets before committing important resources to product development and terminated paths that were not perceived as promising (Table 1).

#### 4.3.2 Increasing the portfolio of exploration methods (Learning 5)

Learning across paths allowed TechLtd to expand its portfolio of available exploration methods (Table 1) and to become more proficient at selecting an appropriate one. A firm that has focused for years on exploitative business strategies will likely lack the exploration methods needed to acquire knowledge about unknown green technologies and markets. These methods go beyond conventional market analysis by including networking activities (such as visiting industry workshops or approaching end users). Conventional market analysis tools typically do not allow a firm to understand the dynamics of emerging green technologies.

On the first path, P1, virtually no market exploration was undertaken and thus no methods were used. On path P2, realizing that relying only on the OEM did not provide them with enough market information, TechLtd initiated a strategic alliance to pursue market exploration. This partnership can be seen as its first new exploration method. Most new exploration methods were introduced on Path P3 after TechLtd realized how important exploration was (see previous section). On Path P3, TechLtd sought for the first time to (a) actively find new customers on its own, (b) attended a workshop to sense the pulse of the emerging industry, and (c) approached leading end users (not customers) with the aim of understanding their needs and so the viability of end‐user business models in a highly volatile the market (see also Table 1). TechLtd learned to carry out in‐depth market analysis before committing important resources to product development. Even though it carried out in‐depth market exploration, path P3 was also eventually terminated because the flywheel markets were not mature enough to yield profits over the short term (see also Wicki & Hansen, 2017). On path P4, some of these methods were discarded because this market was more mature than those on paths P1, P2, or P3. New methods were also introduced, such as the “wait‐and‐see” approach, which consisted of analyzing whether a technology market area is theoretically promising and then waiting to see if it fulfills that potential. Without having made upfront investments, TechLtd periodically surveyed the market for positive signals.

#### 4.3.3 Making exploration more efficient (Learning 6)

Given the high uncertainty that each path involves, it is essential to make exploration more efficient by terminating unviable paths early on and thus saving important resources (in terms of time, personnel, and investment). Improving exploration efficiency allows a firm to increase its overall chances of success, as it is able to “walk down” more paths, increasing the chances that one will lead to success. The findings show an evolution from a random to a more intelligent trial‐and‐error approach, with exploration activities yielding better learning outcomes. This evolution is most visible from Paths P2 to P4 with regard to three elements.

First, the findings show that the effort needed to assess a new business idea decreased in terms of the number of exploration activities needed, the duration of the exploration, and the financial resources involved (Table 1). For instance, Path P3 was terminated after only 5 years, five exploration activities, and about half a million euros, whereas it took top management over 8 years, 11 activities, and three–four million euros to terminate path P2. For Path P4, top management took about 2 years (five activities and also three–four million euros) to adopt a wait‐and‐see approach.

Second, the firm improved at selecting the appropriate exploration methods, making it more efficient at exploration. Indeed, across paths, the use of exploration methods became more targeted and yielded more accurate information. The proficiency at selecting the appropriate method increased along path P3. Fewer methods were used, but they brought exactly the information needed to understand the market dynamics. As the market was not mature, top management terminated this path early on without significant investment (Table 1). TechLtd further improved its ability to select the right method on Path P4, where methods that were only adapted to early‐stage markets were dropped, because they were not useful in this more mature market. The firm's increased proficiency at selecting the right methods also shows that it developed a much better understanding of what information was needed to assess the viability of a new business idea and learned to search for it in a very focused way.

Third, the decision whether or not to pursue an innovation path came earlier and was more pragmatic (Table 1). Interesting but not promising paths—those that might yield commercial success only in a distant and uncertain future—were given less attention. This evolution is visible in the rationales of path termination. On Path P1, the project was continued based on the explicit signal of the main customer who trusted the market. It was only terminated when TechLtd realized it could not protect its intellectual property and thus not capture the value of its innovation. Path P2 was terminated very late, only when it became obvious that there was another way to market the product, which by then was maladapted to market needs. Path P3 was terminated as soon as top management realized that the business model of its end users was not viable. Finally, on Path P4, the wait‐and‐see approach was adopted (instead terminating the project) as soon as TechLtd realized that the market was still too young to be reasonably predicable. From then on, it only invested periodically in market screening. With the exception of the first path (which was relatively simple as the signal came from the customer), it is striking to see how these decisions were taken much earlier on the fourth path compared with the previous two.

## 5 DISCUSSION AND CONCLUSION

### 5.1 Green innovation as path‐based learning processes

This paper empirically examines how green (technology) innovation processes unfold at established firms aiming to seize new business opportunities emerging with sustainable development (Hart et al., 2003). Our findings provide a path‐based learning framework and a fine‐grained longitudinal view of underlying exploration processes (Table 3; Figure 5) and of a typical green innovation journey. Opening up the black box reveals a very different process from the incremental innovation typically observed in established firms with focus on performance (Benner & Tushman, 2003). Far from a planned one‐time action, our research reveals a more complex and nonlinear process that is unfolding over time. It is emergent, with several paths coevolving in parallel, influencing each other, sometimes overlapping, very often intertwined, iterative, and of an unpredictable length. It is an often long—over a decade in the case study, messy, sometimes surprising, discovery process toward an entirely new and unknown business area—much like the entrepreneurial action of starting up a new firm. Our findings corroborate previous research on radical innovation in the nonsustainability context, which found very similar process patterns (Cooper, 1983; Van de Ven et al., 2008).

The findings also reveal that failures play an important role. The unfolding innovation process involves many trials and errors. For the many new things that are learned, numerous mistakes were also made. Failures can therefore not be disassociated from this process; they are a necessary element. Hence, a new perspective on failures is needed, one that considers failures as learning opportunities that are inherent to any creative process where someone discovers and start to learn something new.

The path‐based learning framework offers a new view on green technology innovation processes. It reveals that three types of learning happen along the paths. First, learning happens on‐path, second as path‐initiation learning, and third as cross‐path learning.

#### 5.1.1 On‐path learning

First, a part of the journey is simply to learn an important amount of new things. It is the hard learning work that the firms need to do on each path. At this level, the firm learns about the specificity of green technology and market environments as well as how to use its core competences in emerging environments. It learns what sustainability really means for the firm. As the learning effort required is tremendous and there are probably no shortcuts, firms expect to team up with other actors that do have some of this knowledge (Lavie & Rosenkopf, 2006). The failures at this level relate to learning things that are not needed for the innovation at hand.

#### 5.1.2 Path‐initiation learning

Second, the knowledge generated on each path allows the firm to identify new paths that were not previously accessible. In this process, the size of the innovation space is progressively adjusted and the firm learns to make trade‐offs between a high ambition for sustainability and a more realistic view of what is possible. Related to this learning type, failure consists of investing in an innovation path that is not viable, i.e. walking down a path leading to a dead‐end. However, we concur with previous research that this kind of failure is simply part of an exploratory journey (Van de Ven et al., 2008). Dead‐ends are normal in a trial‐and‐error processes. In fact they even allow new paths to be opened up. Indeed, it is unlikely that a firm would discover new business areas without first exploring one or two unsuccessful ones. Therefore, dead‐ends (and opening up new paths) are simply part of the unfolding journey and should not be seen as failures.

Since dead‐ends are normal and likely more frequent than successful innovations, innovation researchers should further examine the role of this kind of failures in green innovation. For instance, a practical way to better understand their role in this process is to examine if there are typical failures or traps (van Oorschot, Akkermans, Sengupta, & Van Wassenhove, 2013). This research shows at least one trap: The case study firm first aimed at a high impact innovation, before realizing it was too ambitious and eventually finding innovations that better fitted their competences. Knowing more about typical traps may tell us more about the journey toward greater sustainability and help firms speed up their processes.

#### 5.1.3 Cross‐path learning

Third, cross‐path learning allows the firm to evolve from a rather simple and blind approach to a more intelligent form of exploration. The firm becomes faster and more effective at exploration over time, as it comes to understand the need for in‐depth exploration before committing important resources. Moreover, it learns how to better use exploration methods, which allows exploration to be more effective and efficient, increasing the return on exploration (Birkinshaw & Haas, 2016). This evolution is located at the level of believes, cognitive frames, and routines (Tripsas & Gavetti, 2000). It involves abandoning old habits of thought that become limiting for innovation and developing new ones. Turning new thoughts into organizational reality implies creating new routines, which is a tremendous challenge as routines are hard wired into the organization and are thus an important source of inertia (Leonard‐Barton, 1992). New practices that favor a more intelligent exploration may include involving innovation intermediaries (Klewitz, Zeyen, & Hansen, 2012), participating in networks (Halme & Korpela, 2014), or relying on open innovation (Arnold, 2011). It is this evolution that allowed the case study firm to become over time better equipped for exploring emerging business areas.

Related to cross‐path learning, three kinds of failures can be found. First, firms may underestimate the need for upfront exploration, for instance, by believing that there is no need to learn much about a new area. Second, they may use inappropriate exploration methods that do not provide the information needed to pursue or terminate a path. Third, they may not draw the important lessons of previous failures and thus be inefficient at exploration. Efficient exploration processes allow new business areas to be explored quickly, and to decrease the exploration costs of each path. This in turn also allows firms to decrease the high financial risks involved with walking into a dead‐end. As each path costs less, more paths can be explored, thus increasing the ultimate chances for success. It also saves time, which is essential to achieve competitive advantage when other firms are racing for the same emerging markets (Eggers, 2012). Although the failures related with the first two types of learning seem unavoidable, firms can work on this last type of failures.

To be successful at developing green technology, we suggest that firms need to significantly invest in learning “how to explore intelligently.” This appears to be the most important leverage point for firms to successfully navigate the innovation journey. However, learning how to explore more intelligently is an ability that established firms typically lose as they mature and focus on productivity (Benner & Tushman, 2003), and become less entrepreneurial. Therefore, it needs to be continually redeveloped. Even though this is so important, we so far know relatively little about how firms actually develop again the ability to explore intelligently after a long period of exploitation. Hence the ability to do so is perhaps the fundament of what some authors refer to as the green innovation capability (Assink, 2006; Chen, 2008). This seems to be a very interesting avenue for further research.

### 5.2 The complexity of green innovation

The path‐based learning framework also provides empirical insights into the complexity involved with green technology innovation. In the literature, it has often been argued that green technology innovation is more complex than conventional innovation (Seebode et al., 2012; NBS, 2012; Adams et al., 2016). However, the literature features little evidence to support this claim. The fireworks model allowed the study of this unfolding innovation without reducing its complexity and reveals complexity at least at two levels: overall and on each path. First, developing green technology innovation is complex overall because it likely requires several paths to be walked down before one innovation is successfully developed. In one innovation endeavor several individual innovations are embedded, each being very different from the other and bringing their own complexity with it, thus strongly increasing overall complexity.

Second, each path has its own complexity. Our findings show complexity at three levels: First, sustainability markets are more prone to regulatory interventions (Luethi, 2010), at least in the renewable energy context. Regulations change unpredictably, making market evolution more uncertain. This uncertainty increases the difficulty for firms to invest in these markets. Thus, although governmental interventions aim to support market development (Kemp, Schot, & Hoogma, 1998), unpredictable regulation increases entrepreneurial risk and can lead firms to shy away from investment. Second, sustainable technology markets are often still at an early stage of development and typically function differently than mature markets (Suurs et al., 2010). These younger markets are often more volatile, their dynamics less stable, and their evolution less certain. Hence, they are more difficult to analyze. Thus, established firms must first develop appropriate exploration methods to understand the complex dynamics of these markets before entering them. Third, due to the “directional risk”, a green technology innovation idea that was initially considered as having the potential to have a strong sustainability impact may turn out to have less impact in the use phase (Hansen et al. 2009; Paech, 2007). This means that an innovation's sustainability performance needs to be periodically reassessed along the innovation journey. If the firm does not want to compromise its sustainability ambition, this may decrease the size of the innovation space. This would also mean that a number of paths will be terminated because the impact is too low, which may increase the overall costs of the innovation journey.

### 5.3 Management implications

The findings raise important questions about how to successfully manage green technology innovation processes at established firms and make them as ressource efficient and fast as possible. Likely, the failures related with exploring the wrong technologies or markets (relating with T1 and T2) cannot really be avoided. However, the failures related with T3 represent an important leverage point to improve the effectiveness of this process. We therefore focus on two significant ways to improve exploration: first, by fostering an organizational culture that values failures, and second, by developing exploration skills that yield a high return on learning.

First, a safe exploration space can be created within the organization (Raisch, Birkinshaw, Probst, & Tushman, 2009). In this space, exploration failures are not only tolerated but are even valued so as to enrich the exploration process and make it more efficient. Indeed, even though errors are a natural part of trial‐and‐error learning, firms do not necessarily understand their value. In fact, risk and failure adverse organizational cultures are very common (Khanna et al., 2016), possibly because traditional management textbooks emphasize failure avoidance. However, learning from failures is often hampered by important barriers embedded in the social system that are related to adverse psychological reactions to failure (Cannon & Edmondson, 2005). Furthermore, as most firms reward success and punish failure, managers have an additional incentive to disassociate themselves from failure. Therefore, a prerequisite for effective learning from failure is a failure‐friendly organizational space, which according to Sitkin (1992), can be promoted by removing procedural constraints on natural experimentation and by legitimizing “intelligent” failures.

Second, the return on learning from each exploration activity can be increased (Birkinshaw & Haas, 2016). Our findings show a movement from blind to more intelligent trial‐and‐error processes, which progressively yield a higher return from learning. Exploration activities can be purposefully designed as small experiments to yield as much learning as possible (Cannon & Edmondson, 2005; Sitkin, 1992). When an experiment is well‐defined in terms of its expected knowledge gain, it is easier to design further small‐scale experiments to build up, step by step, the knowledge needed for exploration. Another advantage of such a deliberate experiment design is that it allows exploration to be split up into smaller discreet activities. These are easier to manage, also in case of failure. Finally, with numerous small instead of a few large‐scale experiments, the firm can more rapidly assess the viability of a new business idea.

A failure‐friendly space and intelligent trial‐and‐error learning can help overcome the additional complexity that green technology innovation involves and thus represents a good basis for building green exploration capabilities. Given how new and different this is from managing the core business, hiring an external, qualified person to facilitate this exploration is possibly a very good investment for firms aiming to navigate the innovation journey.

### 5.4 Future research

An interesting question for future research is how a firm orients its initial search direction. Noci and Verganti (1999) suggest that firms can use the concept of sustainability as an orientation point in the context of strategic change, and Hart et al. (2003) explain that firms can use green technologies to seize new business opportunities. Our paper shows how exploration might work, but we still know little about how a firm chooses its initial direction of search. Why does a firm develop small wind turbines instead of (green) nanotechnologies? This paper shows that firms may use their core competences as a starting point in their search to create sustainable value in emerging green markets. In this sense, our findings corroborate with insights of the literature on strategic management (Ansoff, 1957) and exploration strategies (Voss & Voss, 2013), which suggest that firms use their core competences to orient their exploration direction. Hence, we suggest that established firms leverage their existing core competences (or assets) to obtain profits in emerging green markets (Kiefer et al., 2018; Shah, Arjoon, & Rambocas, 2016). This approach appears particularly interesting to technology‐driven firms who possess strong core technologies (Kiefer et al., 2018; Taylor & Helfat, 2009), but it is not limited to the technology area. Future research could examine what can trigger such a process. We have, for instance, little clarity whether intrinsic top management motivation—or a set of values (Baumgartner, 2009; Dangelico, 2016; Jenkins, 2009)—is a necessary precondition for engaging in a green technology innovation journey. Or can more conventional (nonsustainability minded) top management teams initiate similar processes? Furthermore, future research could also explore whether firms can also leverage their market position (instead of core competences) for green exploration.

### 5.5 Limitations

The main limitation of this research relates to its research design. We studied a single firm and our results are therefore not simply generalizable. Nevertheless, they are transferable (Guba, 1981) as the learning processes and related outcomes are assumed to be typical of many entrepreneurial SMEs and other organizations (including large ones) in resource‐constrained contexts.

Although we did include external actors in our analysis, a second limitation comes from the firm internal perspective on the learning process. Future research could adopt a broader focus that approaches the innovation process from a network perspective, for instance, using the notion of action‐learning networks (Clarke & Roome, 1999). This complementary perspective would allow a better understanding of the role other actors play in the innovation process. This is particularly relevant in the SME context, where collaboration and networking play a crucial role (Adams et al., 2016). Furthermore, this perspective could also help us better understand how intermediaries—local governments, innovation process facilitators, or consultants—could support a firm on its path of innovating for sustainability (Goodman et al., 2017; Klewitz et al., 2012).

## APPENDIX A

**Table A1:** Empirical data: path origin of detailed learnings in relation to their impact on innovation paths

	Impact on Path 1—FCs	Impact on Path 2—SWTs	Impact on Path 3—FS	Impact on Path 4—WHR
Detailed learnings 1–14 from Path 1 (FCs)	***T1: On‐path learning*** 1. TechLtd learns about FC technology → L1 2. Size of controller needs to be small to fit vehicle applications → L1 3. Strict compliance with many safety standards is needed in the car industry → L1 4. Learns about feed‐in technology (how electricity generated by FCs needs to be transformed to be grid compatible) → L1 5. Discovers characteristics of green markets (numerous small niche markets with different regulations, some are at very early stage or still in formations and thus difficult to predict) → L1 6. Discovers automotive supplier markets → L1 7. Meet actors knowledgeable about feed‐in technology and green markets → L1 8. Norms in car industry (product quality and compliance, need for dedicated department) → L1 9. Automotive markets have high entry barriers due to long supplier selection processed. High risk of important upfront investment → L1 10. High‐volume automotive markets exceed TechLtd's current manufacturing capacity → L1	***T2: Path‐initiation learning*** 4 → L2 5 → L2 7 → L2 11. Mature technologies offer better commercial prospects, unlike those still in emerging as in path P1 → L3 12. Large well‐established markets difficult to penetrate due to important, formalized entry barriers (such as strict norms and supplier selection standards) → L3 13. Market size should not exceed TechLtd's production capacity of <1000 pieces/year → L3 ***T3: Cross‐path learning*** 14. R&D collaboration is very helpful for new product development (to share costs and risks) → L5	***T2: Path‐initiation learning*** 2 → L2 3 → L2 4 → L2 5 → L2 6 → L2 7 → L2 8 → L2 11 → L3 13 → L3 ***T3: Cross‐path learning*** 14 → L5	***T2: Path‐initiation learning*** 4 → L2 5 → L2 7 → L2 11 → L3 12 → L3 13 → L3 ***T3: Cross‐path learning*** 14 → L5
Detailed learnings 15–31 from P2 (SWTs)	n/a	***T1: On‐path learning*** 15. Learns about SWTs and associated technologies → L1 16. Wind turbine management technology (manage highly fluctuating turbine velocity) → L1 17. High efficiency inverter and grid feed‐in technology to minimize conversion losses → L1 18. Meet many SWT market actors → L1 19. Discover global SWT niche market dynamics, and the difference among countries → L1 20. SET markets often come with high product development costs due to national regulation; high costs for market monitoring → L1 21. SET markets are highly dependent on government regulation of renewable energies; require intensive market exploration and adapted exploration methods → L1 22. Early‐stage markets typically involve → L1 – bankruptcy risks – long and difficult trust‐building – lack of professionalism – lack of industry networks – lack of industry lobbies 23. In the SWT market, many poorly functioning turbines create legitimacy problems (that can even impact reputation for component suppliers) → L1	***T2: Path‐initiation learning*** 17 → L2 24. Most inverter and feed‐in components developed for SWT can be reused for FS and WHR → L2 25. Do not go into B2C markets as TechLtd lacks competence to handle large consumer sales and after sales requirements → L3 ***T3: Cross‐path learning*** 20 → L4 21 → L4, L5 22 → L4 26. Industry workshops are good to rapidly meet many market actors and gain market information → L5 27. In SET markets, upfront research is needed about the numerous regulatory frameworks (for instance related with the different feed‐in tariffs in Europe) → L5 28. Approach industry leaders (to understand their business model) allows to rapid have an idea of the market dynamic at play → L5 29. Involve end users in product development (to better understand their needs) → L4, L6 30. Assess viability of the business model of potential clients. Sometimes it even allows to estimate if the overall market is viable; the business model of a waste truck manufacturer showed that flywheels are not ready for automotive applications → L5, L6 31. Rapid prototyping instead of (upfront) full product development → L5, L6	***T2: Path‐initiation learning*** 17 → L2 24 → L2 25 → L3 ***T3: Cross‐path learning*** 20 → L4 21 → L4 26 → L5 27 → L5 29 → L4, L6
Detailed learnings 32–39 from P3 (FS)	n/a	n/a	***T1: On‐path learning*** 32. Learns about FS technology → L1 33. Two main application areas: vehicles and electricity grid → L1 34. Learns to developed and emergency shutdown module in case of electricity cut → L1 35. High complexity as FS can be used for multiple applications in several still emerging markets → L1 36. Large FS fit electricity‐grid, micro FS rather automotive applications → L1 37. FS diffusion dependent on government feed‐in legislation → L1 38. Automotive market: strong dependence on very few market gatekeepers (e.g., automakers, train manufacturers) → L1	***T2: Path‐initiation learning*** n/a ***T3: Cross‐path learning*** 39. A good method is to adopt a “wait‐and‐see” approach in promising markets with high uncertainty about future development → L5, L6
Detailed learnings 40–43from P4 (WHR)	n/a	n/a	n/a	***T1: On‐path learning*** 40. Learn about WHR technology → L1 41. Heat recovery is still a niche market with many different customer applications → L1 42. High search costs for other/new customers in markets with very diverse customer applications → L1 ○ – case by case approach (no standard solutions) ○ – high‐speed solutions only needed in some applications ○ – need to demonstrate advantage of high‐speed solutions to customers 43. WHR market highly dependent on government regulations → L1

Note: FC: fuel cell; FS: flywheel storage; R&D: research and development; SWT: small wind turbine; WHR: waste heat recovery. The detailed Learnings 1–43 are listed and explained in relationship between the Paths P1–P4 they originated on and the Paths P1–P4 they had an impact on. Some detailed learnings have an impact on more than one path. Each detailed learning is related with an “→” to one or more of the six learning outcomes (L1–L6). Some detailed learnings related with two (or more) different learning outcomes. In that case, the table only features their number when they appear the second time (e.g., 2 → L2). Single‐loop learning outcomes that were directly useful for the same path (on‐path) are described under T1. Single and double‐loop learning outcomes useful to initiate new paths (path‐initiation learning) are described under T2, and double‐loop learning outcomes that allowed an improvement of exploration (across path) under T3.

Coding scheme: first order concepts: detailed learnings (1–43); second order themes: learning outcomes (L1–L6); aggregated dimensions learning types (T1–T3).
